# Asthmatic Patients with Vitamin D Deficiency have Decreased Exacerbations after Vitamin Replacement

**DOI:** 10.3390/nu9111234

**Published:** 2017-11-11

**Authors:** Paolo Solidoro, Michela Bellocchia, Ilaria Aredano, Alessio Mattei, Emanuele Pivetta, Filippo Patrucco, Monica Boita, Francesca de Blasio, Luisa Brussino, Giovanni Rolla, Caterina Bucca

**Affiliations:** 1 Azienda Ospedaliero Universitaria Città della Salute e della Scienza, S.C. Pneumologia U, 10126 Torino, Italy; psolidoro@cittadellasalute.to.it (P.S.); michela.bellocchia@libero.it (M.B.); mattei.alessio@virgilio.it (A.M.); filippo_patrucco@hotmail.it (F.P.); 2 Department of Medical Sciences, University of Turin, 10126 Turin, Italy; ilaria.aredano@edu.unito.it (I.A.); emanuele.pivetta@gmail.com (E.P.); monica.boita@libero.it (M.B.); francesca.deblasio1989@gmail.com (F.d.B.); luisa.brussino@unito.it (L.B.); giovanni.rolla@unito.it (G.R.)

**Keywords:** vitamin D deficiency, asthma exacerbations, airway obstruction, vitamin D supplementation

## Abstract

Background: Intervention studies with vitamin D in asthma are inconclusive for several reasons, such as inadequate dosing or duration of supplementation or uncontrolled baseline vitamin D status. Our aim was to evaluate the benefit of long term vitamin D add-on in asthmatic patients with actual vitamin D deficiency, that is a serum 25-hydroxy vitamin D (25-OHD ) below 20 ng/mL. Methods: Serum 25-OHD, asthma exacerbations, spirometry and inhaled corticosteroids (CS) dose were evaluated in a cohort of 119 asthmatic patients. Patients with deficiency were evaluated again after one year vitamin supplementation. Results: 25-OHD was low in 111 patients and was negatively related to exacerbations (*p* < 0.001), inhaled CS dose (*p* = 0.008) and asthma severity (*p* = 0.001). Deficiency was found in 90 patients, 55 of whom took the supplement regularly for one year, while 24 discontinued the study and 11 were not adherent. Patients with vitamin D deficiency after 12 months supplementation showed significant decrease of exacerbations (from 2.6 ± 1.2 to 1.6 ± 1.1, *p* < 0.001), circulating eosinophils (from 395 ± 330 to 272 ± 212 10^6^/L, *p* < 0.001), and need of oral CS courses (from 35 to 20, *p* = 0.007) and improvement of airway obstruction. Conclusions: Asthma exacerbations are favored by vitamin D deficiency and decrease after long-term vitamin D replacement. Patients who are vitamin D deficient benefit from vitamin D supplementation.

## 1. Introduction

In the last decades, the prevalence of asthma in the general population has been increasing [1], as well as the recognition of vitamin D deficiency and insufficiency [2]. Asthma and vitamin D deficiency recognize multiple common risk factors such as industrialization, poor diet, obesity, dark skin pigmentation, winter season, and high latitude [3,4,5,6,7,8,9]. Based on these evidences, Litonjua and Weiss [10] formulated the hypothesis that “vitamin D deficiency is to blame for the asthma epidemic”. A growing body of literature supports the concept that vitamin D is involved in the pathogenesis of asthma, sinusitis, and allergic rhinitis, since vitamin D regulates a broad range of immune and non-immune cells, and modulates innate and adaptive immune response and cell growth [11,12,13]. Nevertheless, the relationship between asthma and vitamin D is still debated. Several extensive and comprehensive reviews are available on the relationship of vitamin D and respiratory health [14,15,16]. Kerley et al. [14] analyzed most epidemiologic, case-control, and cross-sectional studies on vitamin D status and asthma, highlighting the association of vitamin D deficiency with airway hyperresponsiveness, decreased pulmonary function, worse asthma control, and decreased response to standard antiasthma therapy [17,18,19]. Luo et al. [15] rigorously analyzed all the intervention studies with vitamin D published over the last seventy years: only seven trials responded to the selection criteria, all showing no benefit of adding vitamin D to asthma controllers on exacerbations, airway obstruction, and symptoms. Martineau et al. [16] conducted a meta-analysis restricted to double-blind, placebo-controlled trials of at least 12 weeks duration on the effect of vitamin D on exacerbations requiring systemic corticosteroids, concluding that there is a need for research focusing on the role of age, asthma severity, and vitamin D status on the response to supplementation. Actually, most studies are biased by limitations, such as small case number, too short supplementation, patients’ heterogeneity in respect to asthma severity and baseline vitamin D status [20,21,22,23]. In terms of age, the effectiveness of vitamin D supplementation in decreasing asthma exacerbations seems to be better in children [20,21] even in preschool time [23].

In our opinion, interventional studies to assess the role of vitamin D supplementation in asthma should focus on patients with actual vitamin deficiency and should evaluate the effect of long-term supplementation. The view that vitamin D can only be effective in those who really are deficient is supported by a recent systematic review and meta-analysis by Martineau et al. [24] about the prevention of respiratory infections.

The aim of the present study was to investigate vitamin status of asthmatic patients in relation to exacerbations and to evaluate if long term vitamin D supplementation for one year in patients with vitamin deficiency (25-OHD below 20 ng/mL) improves asthma control, in terms of exacerbations, corticosteroid consumption, and airway obstruction.

To these aims, we firstly performed a cross-sectional examination of a cohort of 119 asthmatic patients, who had at least one year regular follow-up at our respiratory clinic, and who were taking no vitamin D supplementation.

Thereafter, we invited the patients who were D deficient to attend a longitudinal intervention study on the effect of one year supplementation with vitamin D.

The study started and ended in the late autumn-winter season when sun exposure is lowest at our latitude.

## 2. Materials and Methods

All the consecutive adult patients with bronchial asthma of any severity presented to the Asthma Clinic, Pneumology Department, of the University Hospital “Città della Salute e della Scienza”, Turin, Italy, for a scheduled visit during the period October-March, who had quarterly visits in the last year, were enrolled into the study.

Asthma was diagnosed and classified according to the Global Initiative for Asthma (GINA) strategy [25], based on symptoms plus evidence of airway obstruction with bronchodilator reversibility or of bronchial hyperresponsiveness to methacholine challenge. Exclusion criteria were acute asthma exacerbations in the month preceding the enrolment, current malignancy, other severe respiratory and systemic diseases, and treatment with vitamins and dietary supplements in the last year.

The study was conducted in accordance with the amended Declaration of Helsinki, and was approved by the Institutional Review Board “Comitato Etico Interaziendale A.O.U. San Giovanni Battista (CEI N. 415). TRIAL REGISTRATION: Trial registry: ClinicalTrials.gov; Unique Identifying number: NCT02661191; Registered 28 January 2016. Informed consent was obtained from each patient.

The study was in two phases:

1. a cross-sectional retrospective study on a cohort of asthmatic patients
2. intervention study in patients with vitamin D deficiency, consisting in one year vitamin D supplementation.

**Figure 1:**
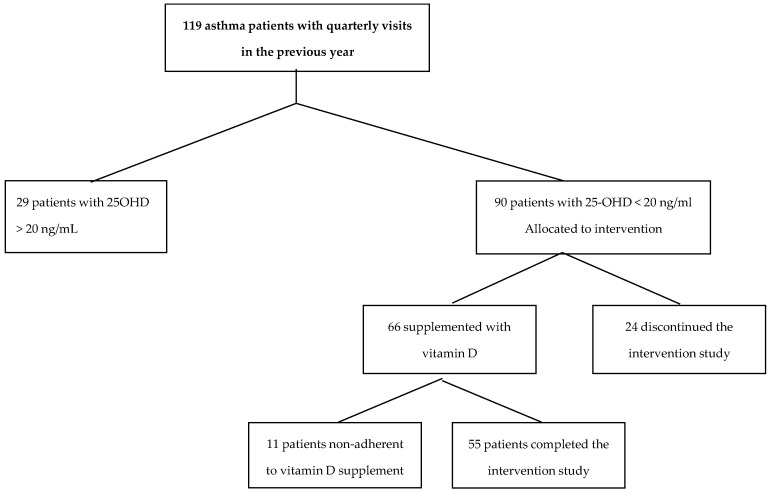
Consort flow diagram showing the design and conduct of the study.

A consort flow diagram outlines the design and conduct of the study, see Figure 1.

### 2.1 Phase 1–Cross-Sectional Study

At the enrolment visit, patients underwent clinical examination and recording of symptoms, smoking habits, medication use, atopy, comorbidities, lung function tests, fractional exhaled nitric oxide (F_E_NO) and venous puncture for laboratory tests. Subjects were classified as current, ex- and never-smokers, according to self-reported smoking history. Body mass index (BMI) was calculated as the ratio weight by squared height (kg/m^2^).

Atopy was defined by the presence of at least one positive skin prick test, according to the European Academy of Allergy and Clinical Immunology (EAACI) consensus on allergy testing [26]. Comorbidities were recorded on the basis of prior diagnosis and current treatment for: systemic arterial hypertension, diabetes, anxiety and/or depression, chronic kidney disease, cerebrovascular disease, osteoporosis, and obstructive sleep apnea (OSA).

Lung function tests were measured using the Baires System (Biomedin, Padua, Italy). The values of slow vital capacity (VC), forced expiratory volume in one second (FEV_1_), and the FEV_1_/VC% ratio, were used as markers of airway patency. VC and FEV_1_ were expressed either as absolute values or as percent of the predicted value [27]. Reversibility of airway obstruction was established if FEV_1_ increased by 12% from baseline or by 200 mL following inhalation of albuterol 400 μg [25]. Bronchial responsiveness was assessed, when appropriate, by methacholine challenge, according to American Thoracic Society (ATS) guidelines [28].

F_E_NO was measured according to ATS/European Respiratory Society (ERS) recommendations [29], using a NO electrochemical analyzer (Hypair, Medisoft, Sorinnes, Belgium).

Laboratory tests included circulating eosinophil count and nutritional assessment, that is: serum levels of ferritin, folic acid and vitamin B12, measured using the chemiluminescent micro-particle immunoassay (Architect System, Abbott diagnostic division, Longford, Ireland), and of 25-hydroxy vitamin D (25-OHD), measured by the Radioimmunoassay (RIA) method (25OH Vitamin D total-Ria-CT Kit, DIA source ImmunoAssay S.A., Louvain, Belgium). Vitamin D levels were regarded as normal (equal or over 30 ng/mL), insufficient (range 20 to 29 ng/mL), deficient (range 10 to 19 ng/mL), and severely deficient (below 10 ng/mL) [2].

The medical record of each patient was collected and reviewed retrospectively, to gain information relative to lung function, therapy and number of acute asthma exacerbations throughout the year prior to enrolment in the study. Asthma medications included inhaled long acting beta-agonists (LABA), and antimuscarinic agents (LAMA), inhaled corticosteroids, and oral leukotriene receptor antagonists. Inhaled corticosteroids dose was categorized on the basis of clinical comparability to beclomethasone dose, as suggested in the GINA strategy [25], that is: 1= no inhaled corticosteroid, 2 = low (200–500 mcg), 3 = medium (>500–1000 mcg), 4 = high (>1000 mcg).

Asthma exacerbations were defined according to the ATS/ERS joint statement [30] on the basis of unscheduled physician visits for acute or subacute worsening of respiratory symptoms, associated with airflow obstruction, requiring changes or higher doses of medications, need for oral corticosteroid or antibiotic, and/or hospitalization. The number of patients per year who needed one or more oral corticosteroid course to treat an exacerbation of asthma symptoms was recorded.

### 2.2 Phase 2–Intervention Study

All patients found to have vitamin D deficiency (25-OHD below 20 ng/mL) were invited to participate in the intervention study with vitamin D add-on for one year, along with their regular treatment. The same asthma medications schedule was maintained throughout the study. This phase consisted of a starting dose of intramuscular cholecalcipherol 100,000 IU administered during the clinic visit, followed by self-administered oral cholecalcipherol 5000 IU (20 drops of a 10,000 IU/mL oral solution) weekly, plus 400 IU daily (chewable tablet containing also calcium carbonate 1500 mg) for one year. The dose was established on the basis of the 2011 Endocrine Society guidelines [31]. Adherence to treatment was stressed at each visit by reminding of taking vitamin D drops and pills. During treatment, spirometry, F_E_NO value, as well as the occurrence of exacerbations were assessed every three months. Measurement of 25-OHD and circulating eosinophils was made only at the end of the supplementation year.

Data recorded in the year before vitamin D supplementation (baseline) were compared to those observed during vitamin D add-on (outcome).

### 2.3 Statistical Analysis

All statistics were performed with SPSS Statistical Package software, V 21 (SPSS, Chicago, IL, USA). A descriptive analysis of all variables was done. The normality of variable distribution was assessed by the Kolmogorov-Smirnov test.

To investigate the relationship between the baseline 25-OHD concentration and some baseline features of the population, General Linear Models were applied, with a dependent variable being either 25-OHD or the number of exacerbations, and as covariates general characteristics, nutrition parameters, FEV_1_ (% predicted), FEV_1_/VC%, F_E_NO, inhaled corticosteroid score, and comorbidities. Linear regression models were used because the dependent variables (i.e., 25-OHD and number of exacerbations) were continuous. Most of the independent variables (such as lung function tests, corticosteroid therapy, comorbidities) were chosen on the basis of literature data regarding factors causally related with vitamin D deficiency.

Linear regression analysis was used to evaluate the relationship of 25-OHD with asthma severity (GINA class) and exacerbation number.

The comparison between patients who did and those who did not receive vitamin D add-on was performed by the ANOVA test for parametric variables and with the Pearson’s chi-square test for categorical variables; when the value in any cell was below 5, the Fischer exact test was used; in case of a greater than 2 × 3 contingency table, the chi-square with Yates correction was used. The comparison between data pre and post vitamin D supplementation was performed with the Student’s T test for paired data, or the Mann-Whitney-test. To this aim, the median of the values recorded every three months of F_E_NO, VC, FEV_1_ and FEV_1_/VC% before treatment were compared to those recorded during supplementation.

The results were considered statistically significant if the *p* value was below 0.05.

## 3. Results

### 3.1 Cross-Sectional Study

The studied population included 119 asthmatic patients, 94 (79%) women and 25 (21%) men. The general characteristics of the subjects are reported in Table 1, and the prevalence of comorbidities in the Table S1. The values of 25-OHD were abnormal in most patients (111, 92%), and in 90 (76%) were below 20 ng/mL. Most subjects (70%) were atopic, and had moderate to severe asthma, with frequent exacerbations; seven patients (6%) had undergone at least one hospital admission for asthma exacerbation. The most common comorbidities were rhinitis, rhinosinusitis and systemic arterial hypertension.

**Figure 2:**
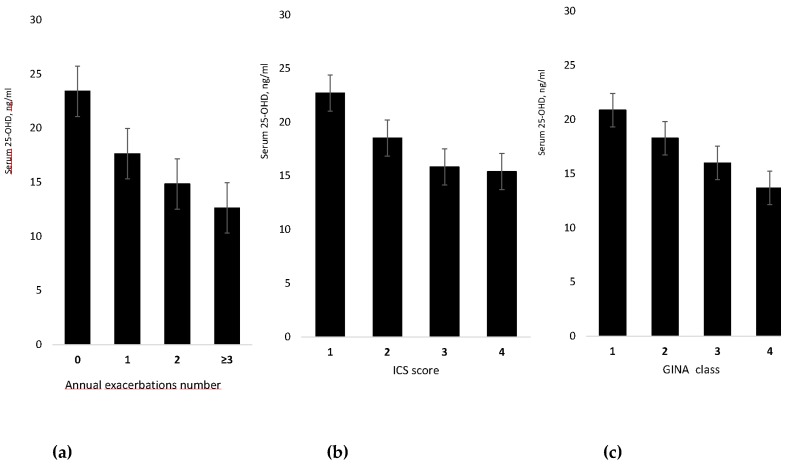
Mean value (with standard deviation) of serum level of 25-hydroxy vitamin D by annual exacerbation number (**a**), score of inhaled corticosteroids dose (**b**), and GINA class of asthma severity (**c**).

Data analysis showed that serum 25-OHD level was significantly and inversely related to the number of annual exacerbations (*R* = 0.474, *p* < 0.001). Moreover, 25-OHD was inversely related to inhaled corticosteroid score (*R* = 0.336, *p* = 0.008) and to GINA asthma severity class (*R* = 0.290, *p* = 0.001), see Figure 2, and was directly related to the median annual value of FEV_1_% predicted (*R* = 0.25, *p* = 0.005) and of FEV_1_/VC% (*R* = 0.26, *p* = 0.004).

General Linear Model analyses on exacerbations in the past year showed that exacerbation number was significantly associated with the present level of 25-OHD (*p* = 0.005) and, consequently with treatment with oral corticosteroids (*p* = 0.001). Lung function, comorbidities and nutrients other than 25-OHD (i.e. ferritin, folic acid and vitamin B12) had no effect on exacerbations. The results of the analyses are reported in the Supplemental Tables: Tables S2, S3 and S4. General Linear Model analyses on vitamin D showed that 25-OHD deficiency was associated with exacerbations (*p* < 0.001), with rhinosinusitis (*p* = 0.015) and with osteoporosis (*p* = 0.010). The results of the analyses are reported in the Supplemental Tables: Tables S5 and S6. Serum 25-OHD concentration in patients with and without rhinosinusitis was 15.3 ng/mL and 17.6 ng/mL respectively, and in patients with and without osteoporosis it was 13.3 ng/mL and 17.9 ng/mL, respectively.

### 3.2 Phase 2

The second phase of the study consisted in the evaluation of the effect of one year vitamin D supplementation in deficient patients (25-OHD < 20 ng/mL) on asthma exacerbations. Out of the 90 deficient patients, 55 patients (61%) remained on treatment for the entire study duration, 24 (27%) discontinued the study (six changed their residence, nine did not attend the starting visit nor even the subsequent appointments, and nine voluntarily discontinued the study at the second appointment after receiving the initial vitamin D injection). Moreover, 11 patients (12%) admitted to be poorly adherent, although at each visit they were reminded of taking vitamin D. Thus, at the end of the intervention year, their 25-OHD was similar to that recorded at enrolment. Baseline values (obtained at the beginning of 12 month supplementation period) of patients without vitamin D deficiency (25-OHD over 20 ng/mL) (group 1), of those with deficiency who discontinued the study or were not adherent to supplementation (group 2) and of those with deficiency who completed the study (group 3) are shown in Table 2. Group 3 patients were older, had more severe asthma, more exacerbations, higher prevalence of rhinosinusitis, systemic arterial hypertension and osteoporosis, and received higher dose of inhaled corticosteroid; half of them was in chronic treatment with leukotriene receptors antagonist and one third with LAMA. Baseline 25-OHD was similar in deficient patients adherent and non-adherent to supplementation. The anti-asthma therapy was not changed compared to the pre-supplementation year.

**Figure 3:**
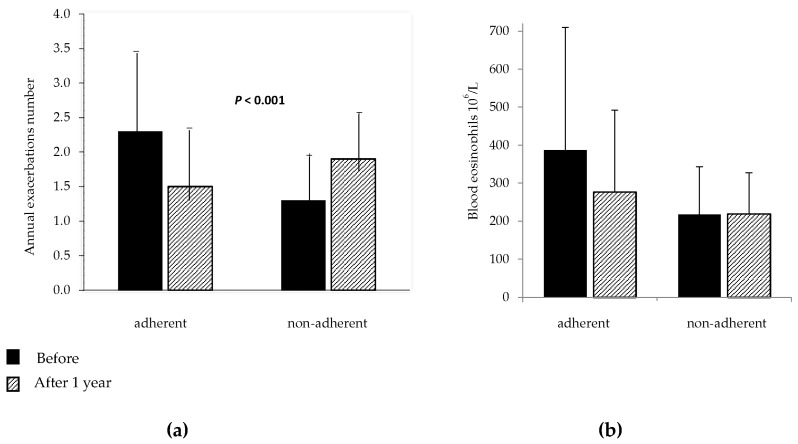
Mean values with standard deviation of annual exacerbations number. (**a**) and blood eosinophils count; (**b**) before and after vitamin D supplementation in patients adherent and non-adherent to vitamin D supplementation.

**Figure 4:**
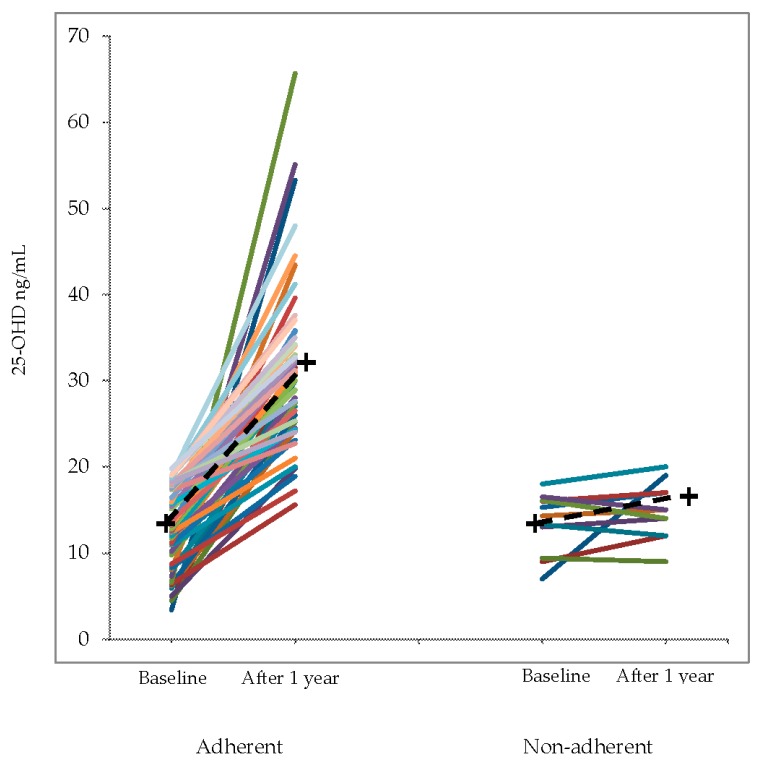
Individual values of 25-OHD before and after vitamin D supplementation in adherent and non-adherent patients. The crosses and the dashed lines represent the mean change.

**Figure 5:**
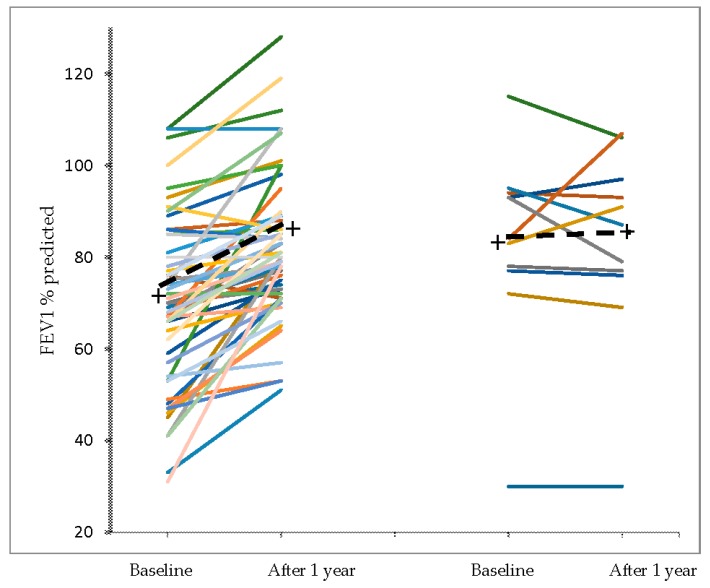
Individual values of the median annual FEV_1_ (% of predicted) before and after vitamin D supplementation in adherent and non-adherent patients. The crosses and the dashed lines represent the mean change.

The results obtained after one-year vitamin D add-on in the 55 patients who completed the study and in the 11 patients who were not adherent to treatment are shown in Table 3 and in the Figure 3, Figure 4 and Figure 5.

In the Figure 4 and Figure 5 are shown, respectively, the individual values of 25-OHD and of median annual FEV_1_ value (% of predicted) before and after vitamin D supplementation in adherent and non-adherent patients.

As expected, in the adherent patients the levels of 25-OHD were significantly increased and most subjects achieved values within the normal range (25-OHD ≥ 30 ng/mL). During supplementation, the number of asthma exacerbations decreased significantly and a smaller number of patients needed oral corticosteroid bursts. The median annual values of VC, FEV_1_ and FEV_1_/VC% were significantly higher than in the year before supplementation. Blood eosinophils decreased significantly after treatment, while F_E_NO showed no significant change. By contrast, in the 11 patients non-adherent to treatment, no significant change was found in annual exacerbation number FEV_1_, FEV_1_/VC%, eosinophils and, as expected, 25-OHD.

## 4. Discussion

The results of this study meet the primary aims of the study, in that they show that vitamin D deficiency was strongly associated with frequent asthma exacerbations and that vitamin supplementation to deficient patients favorably influenced the course of asthma, resulting in less exacerbations, less need of oral corticosteroids bursts, and improved airway obstruction.

The results of the cross-sectional study showed that most asthmatic patients examined (93%) had an inadequate vitamin D status, with 17.5% of patients having insufficiency (25-OHD < 30 ng/mL) and 75.5% having deficiency (<20 ng/mL).This finding does not surprise, since vitamin D deficiency has been frequently reported in asthmatic patients [10,11,15,16]. Moreover, 25-OHD was assessed in winter, when levels are supposed to be lowest [32] especially at the latitude of our city, beyond the 45th parallel [33].

As hypothesized, low vitamin D was associated with greater asthma severity, as suggested by the significant negative relationship of 25-OHD with number of exacerbations, GINA class of asthma severity [25] and inhaled corticosteroid dose (see Figure 2), and by the significant positive relationship with the FEV_1_ value. The association of vitamin D insufficiency with asthma exacerbations and severity has been reported by several trials in children and adults [10,17,18,19,21,23,34,35,36,37]. In Puerto Rican children, Brehm et al. [34] found that the influence of low vitamin D on exacerbations was independent of racial ancestry, atopy, markers of disease severity, and asthma control.

As shown in the supplemental Tables, in our cohort 25-OHD was the only factor significantly related with exacerbations, while age, sex, BMI, cigarette smoking, spirometric variables, F_E_NO, asthma therapy (including inhaled corticosteroids and bronchodilators, and oral leukotrienes-receptor antagonist), and nutrients other than vitamin D, had no significant influence.

In agreement with prior reports [17,35,38,39,40], we found that low 25-OHD was associated with the need for a higher corticosteroid dose, a finding that can be attributed to vitamin D deficiency causing greater asthma severity and need for heavier treatment (see Figure 2). However, according to recent observations the matter seems more complex. Sutherland et al. [38] observed that vitamin D deficiency impairs glucocorticoid response, independent of treatment, and Ian et al. [39] found that low vitamin D induces corticosteroid resistance. These findings, along with the demonstration by Gupta et al. [40] that airway smooth muscle mass is inversely related to 25-OHD levels, suggest that vitamin D deficiency may contribute to asthma severity by favoring steroid resistance and airway remodeling.

The association of low vitamin D with airway obstruction is supported by several studies, not only in asthmatic patients [34,35,36,37,38,40,41], but also in unselected populations [42,43].

Interestingly, the only comorbidity associated with vitamin D deficiency, apart from the predictable osteoporosis, was chronic rhinosinusitis, a disease that shares clinical and biologic features with asthma. A recent review of the scarce literature on the subject by Stokes and Rimmer [44] concludes that low vitamin D levels are associated with severe chronic rhinosinusitis with polyps, and suggests a potential for therapy with vitamin D.

The results of the interventional study agree with the main goal of the study, indicating that one year vitamin D supplementation to asthmatic patients with vitamin deficiency was associated with a significant disease improvement, consisting in decreased number of exacerbations, reduced need for oral corticosteroid course, and increased median annual values of VC, FEV_1_ and FEV_1_/VC%. Interestingly, a significant decrease in circulating eosinophils was observed, which, according to Price [45], might be related to the reduction of asthma exacerbations. To our knowledge, a decrease in eosinophils by vitamin D has only been reported in sputum [46] but not in peripheral blood.

There are only a few studies on the effect of vitamin D supplementation in adult asthmatic patients with vitamin deficiency. The vitamin D supplementation dose given in these studies, made the appropriate proportions, was similar or even higher to that we gave to our patients (506,000 IU). In the randomized, double-blind, parallel, placebo-controlled VIDA trial, Castro et al. [23] found no effect of 884,000 IU vitamin D on exacerbations, apart from a small reduction in the maintenance dose of inhaled corticosteroids. However, some considerations should be made before one could draw any conclusion on the ineffectiveness of vitamin D supplementation. Compared to our patients, the participants of VIDA study had actually a better initial vitamin D status, only 66% being vitamin D deficient (<20 ng/mL), had better lung function (mean FEV_1_ 80.7% predicted), and fewer of them needed systemic corticosteroids courses (33.3 patients/year versus 63.6 in our study). Moreover, the supplementation/examination period lasted only 28 weeks and was not controlled for season, so that some patients, including controls, were examined in the sunny months.

Our study has several limitations. First of all, by being an observational cohort study, including consecutive patients uncontrolled for asthma severity, the resulting absence of randomization may be a source of relevant biases [47]. However, all the asthma severity classes, apart from intermittent asthma, were well balanced in our series, see Table 1. Moreover, observational studies reflect the “real world” and can provide clinically relevant information, not always obtained by randomized clinical trials, in terms of patients’ heterogeneity and medical interventions [48]. Second, the cross-sectional study relied on retrospective documentation. However, only those patients with available quarterly records from the previous year were enrolled, adding consistency to the results. Third, the relationship of 25-OHD with the annual trend of parameters and exacerbations number is based on the assumption that vitamin D levels truly mirrored the previous year value trend. Finally, the intervention study was open and lacked a control group. However, randomizing deficient patients to supplementation means that some would remain deficient for a whole year, which seems unethical. Moreover, the 11 patients who were compliant to the survey but were poorly adherent to vitamin D supplementation could represent an unintentional control group. Although the number is too small to draw conclusions, these patients showed no significant difference in exacerbations episodes and lung function tests in the year before and after enrolment.

Finally, the number of patients who discontinued the study is relevant. Interestingly, as compared to the adherent patients (see Table 2), these patients were younger, had less severe asthma and milder airway obstruction, and therefore could be less motivated to participate in the study

## 5. Conclusions

The results of this study indicate that in asthmatic patients, vitamin D deficiency favors exacerbations and affects asthma control.

The finding that in patients with vitamin D deficiency, long-term supplementation, in addition to standard asthma medication, reduces exacerbations, suggests that screening of vitamin levels and restoring normal amounts could be a simple strategy for improving asthma management. Vitamin D supplementation should be aimed at achieving and maintaining levels of 25-OHD over 30 ng/mL throughout the year. In patients with deficiency, a daily dose of 1500–2000 IU should be sufficient for the purpose of raising the blood level of 25-OHD above 30 ng/mL [31].

## Figures and Tables

**Table 1:** General characteristics of the 119 patients.

Women/Men, *n*	94/25
Age, years (range)	52 (13–80)
Never smokers, *n* (%)	94 (79)
Ex-smokers, *n* (%)	11 (9)
Current smokers, *n* (%)	14 (12)
Vitamin D status:	
Normal, *n* (%)	8 (7)
insufficient, *n* (%)	21 (17)
deficient, *n* (%)	90 (76)
BMI, kg/m^2^	24 ± 0.4
Atopy, *n* (%)	83 (70)
VC% predicted	86 ± 1
FEV_1_% predicted	78 ± 2
FEV_1_/VC%	67 ± 1
F_E_NO, ppb	42.9 ± 3.9
GINA class:	
1	11 (9)
2	36 (30)
3	41 (35)
4	31 (26)
N. exacerbations/year	
0	12 (10)
1	37 (31)
2	31 (26)
≥ 3	39 (33)
ICS dose:	
No, *n* (%)	8 (7)
Low, *n* (%)	20 (17)
Medium, *n* (%)	30 (25)
High, *n* (%)	61 (51)
ICS alone, *n* (%)	3 (3)
ICS + LABA, *n* (%)	108 (90)
ICS + LABA + LAMA, *n* (%)	23 (19)
Oral corticosteroid, *n* (%) (*)	55 (46)

(*) Number of patients who needed one or more oral corticosteroid course per year. Abbreviations: BMI = Body Mass Index; VC = Vital Capacity; FEV_1_ = Forced Expiratory Volume in 1 Second; F_E_NO = Fractional Exhaled Nitric Oxide; GINA = Global INItiative for Asthma; ICS = Inhaled Cortico Steroid; LABA = Long Acting Beta Agonist; LAMA = Long Acting anti Muscarinic Agent.

**Table 2:** Comparison among patients with and without vitamin D deficiency at the beginning of 12 month supplementation period.

	Group 1 25-OHD > 20 ng/mL	Group 2 25-OHD < 20 ng/mL 24 Discontinued, 11 Non-adherent	Group 3 25-OHD < 20 ng/mL Adherent to Vitamin D Supplement	*p*
Number	29	35	55	
Age (years)	44 (38–50) ^c^	50 (45–56) ^c^	57 (54–61)	0.001
Men *n* (%)	6 (21)	7 (20)	12 (22)	0.97
BMI (Kg/m^2^) mean (95% CI)	24 (22–25)	24 (22–26)	25 (24–26)	0.29
Smokers *n* (%)	4 (14)	1 (3)	9 (16)	0.14
GINA				
1. Intermittent *n* (%)	5 (17)	4 (11)	2 (4)	
2. Mild persistent *n* (%)	14 (48)	13 (37)	9 (16)	0.002
3. Moderate persistent *n* (%)	8 (28)	10 (29)	23 (42)	
4. Severe persistent *n* (%)	2 (7)	8 (23)	21 (38)	
VC% pred. mean (95% CI)	94 (87–101) ^c^	87 (83–91) ^c^	80 (77–84)	<0.001
FEV_1_% pred. mean (95% CI)	88 (81–96) ^c^	82 (75–89) ^c^	70 (65–75)	<0.001
FEV_1_/VC% mean (95% CI)	71 (67–75) ^c^	70 (65–75) ^c^	63 (59–66)	0.007
F_E_NO ppb mean (95% CI)	47 (22–72)	40 (36–52)	45 (20–60)	0.83
Exacerbations *n* mean (95% CI)	1 (0.6–1.5)	2.2 (1.7–2.6) ^a^	2.6 (2.2–2.9) ^a,b^	<0.001
Eosinophils 10^6^/L mean (95% CI)	320 (190–460)	330 (220–440)	380 (290–470)	0.66
Vitamin D, ng/mL mean (95% CI)	26.6 (25–29) ^b,c^	13.6 (12–15) ^a^	13.0 (12–14) ^a^	<0.001
Atopy *n* (%)	22 (76)	21 (60)	40 (73)	0.31
Rhinitis *n* (%)	22 (76)	25 (71)	42 (76)	0.86
Rhinosinusitis *n* (%)	8 (28)	17 (49)	32 (58)	0.03
Hypertension *n* (%)	11 (38)	13 (37)	37 (67)	0.005
Heart disease *n* (%)	10 (37)	10 (29)	25 (46)	0.25
Depression *n* (%)	7 (26)	12 (35)	9 (16)	0.15
Thyroid disease *n* (%)	8 (30)	5 (15)	16 (29)	0.25
OSA *n* (%)	3 (11)	1 (3)	4 (7)	0.51
COPD *n* (%)	7 (26)	6 (18)	8 (14)	0.55
Osteoporosis *n* (%)	4 (15)	7 (21)	26 (47)	0.002
GERD *n* (%)	8 (30)	9 (27)	22 (42)	0.29
ICS + LABA *n* (%)	24 (83)	32 (91)	55 (100)	0.009
LAMA *n* (%)	6 (21)	5 (14)	18 (33)	0.12
LTRA *n* (%)	4 (14)	7 (20)	28 (51)	<0.001
Oral corticosteroid *n* (%) (*)	7 (25)	13 (37)	35 (64)	0.001
ICS: No *n* (%)	5 (17)	3 (9)	0 (0)	0.04
Low *n* (%)	7 (24)	9 (26)	4 (7)	
Medium *n* (%)	6 (21)	8 (23)	16 (29)	
High *n* (%)	11 (38)	15 (27)	35 (64)	

Group 1: patients with 25-OHD ≥ 20 ng/mL; Group 2: patients with 25-OHD < 20 ng/mL who discontinued the study or were non-adherent to vitamin D supplementation; Group 3: patients with 25-OHD < 20 ng/mL adherent to vitamin D supplementation; ^a^ = significantly different from group 1; ^b^ = significantly different from group 2; ^c^ = significantly different from group 3; (*) number of patients who needed one or more oral corticosteroid course per year. Abbreviations: BMI = Body Mass Index; VC = Vital Capacity; FEV_1_ = Forced Expiratory Volume in 1 Second; F_E_NO = Fractional Exhaled Nitric Oxide; GINA = Global INItiative for Asthma; ICS = Inhaled Cortico Steroid; LABA = Long Acting Beta Agonist; LAMA = Long Acting anti Muscarinic Agent; LTRA = LeukoTriene Receptors Antagonist.

**Table 3:** 25-OHD, exacerbations, lung function tests and F_E_NO before and after vitamin D supplementation in the 55 patients adherent and in the 11 patients non-adherent to vitamin D supplementation.

Variable	Before Supplement	After Supplement	Mean Difference (95% CI)	*p* Value
**Patients adherent**	55	55		
25-OHD, ng/mL	13 (4.8)	31.7 (9.3)	18.7 (15.9–21.4)	<0.001
Asthma Exacerbations, *n*	2.6 (1.2)	1.6 (1.1)	−1 (−0.7–−1.3)	<0.001
Oral corticosteroid, *N* (%) (*)	35 (64)	20 (40)		0.007
FEV_1_, l	1,86 (0.7)	2.11 (0.8)	0.25 (0.16–0.35)	<0.001
FEV_1_/VC%	63 (13)	67 (9)	4 (1–6)	0.002
Blood eosinophils 10^6^/L (**)	390 (330)	270 (200)	−120 (−50–−200)	0.002
**Patients non-adherent**	11	11		
25-OHD, ng/mL	13.7 (3.6)	15.7 (3.9)	1.9 (1.0–4.8)	0.18
Asthma Exacerbations, *n*	2.0 (0.9)	2.0 (0.9)	0 (−0.7–0.7)	1.00
Oral corticosteroid *N* (%) (*)	5 (45)	6 (55)		1.00
FEV_1_, l	2.26 (0.75)	2.19 (0.77)	0.07 (−0.25–0.12)	0.80
FEV_1_/VC%	69 (16)	69 (17)	0.1 (−3.2–3.4)	0.94
Blood eosinophils 10^6^/L	270 (210)	260 (180)	−6 (−56–40)	0.81

Values are given as mean with Standard Deviation; VC = Vital Capacity; FEV_1_ = Forced Expiratory Volume in 1 Second; are expressed as percent of the predicted value; (*) Number of patients who needed one or more oral corticosteroids course per year; (**) assessed in 52 out of the 55 patients; In Figure 3 are shown mean values with standard deviation of exacerbations and blood eosinophils; before and after vitamin D supplementation in adherent and non- adherent patients.

## Supplementary Materials

The following are available online at www.mdpi.com/2072-6643/9/11/1234/s1, Table S1. Prevalence of comorbidities in the 119 asthma patients, Table S2. Results of the General linear model analysis on the relationship of exacerbations with patients’ characteristics, lung function tests and F_E_NO. Age, sex, BMI, cigarette smoking, spirometric variables and F_E_NO had no significant influence on exacerbation rate, Table S3. Relationship of asthma exacerbations with patients’ characteristics, and asthma therapy. Anti-asthma treatment with inhaled corticosteroids (ICS), long acting beta-agonist (LABA) and anti-muscarinic (LAMA),and oral leukotrienes-receptor antagonist (LTRA) had no significant influence on exacerbations. The only factor significantly related to the number of exacerbations was the need of oral corticosteroid course, Table S4. Relationship of asthma exacerbations with nutritional status. The only nutritional factor significantly related to the number of exacerbations was vitamin D (25-OHD). Other nutrients had no significant influence on exacerbations The only nutritional factor significantly related to the number of exacerbations was vitamin D (25-OHD). Other nutrients had no significant influence on exacerbations, Table S5. Relationship of vitamin D with exacerbations, lung function tests and ICS dose. Vitamin D was significantly negatively related with the number of exacerbations, but was independent of lung function tests and dose of inhaled corticosteroids (ICS), Table S6. Relationship of vitamin D with comorbidities. The only comorbidities related with vitamin D were rhinosinusitis and osteoporosis. Other comorbidities, in particular concomitant COPD, had no significant influence on the levels of 25-OHD.
