# Supplementary material for: Asthmatic Patients with Vitamin D Deficiency have Decreased Exacerbations after Vitamin Replacement
Source: Nutrients. 2017 Nov 11;9(11):1234. doi: 10.3390/nu9111234 (PMC5707706; doi:10.3390/nu9111234)
Supplement: Supplementary file 1 [file nutrients-09-01234-s001.docx]

Table S1. Prevalence of comorbidities in the 119 asthma patients

| Comorbidity | N (%) |
| --- | --- |
| Rhinitis | 89 (74.8) |
| Rhinosinusitis | 57 (47.9) |
| Systemic arterial hypertension | 61 (51.6) |
| Heart disease | 45 (37,8) |
| GERD | 39 (32.8) |
| Thyroid disease | 29 (24.4) |
| Anxiety/depression | 28 (23.5) |
| COPD | 21 (17.6) |
| Osteoporosis | 37 (31.1) |
| OSA | 8 (06.7) |

TABLE S2

Results of the General linear model analysis on the relationship of exacerbations with patients’ characteristics, lung function tests and F_E_NO. Age, sex, BMI, cigarette smoking, spirometric variables and F_E_NO had no significant influence on exacerbation rate.

Dependent variable: number of exacerbations per year

| Source | Type III Sum of Squares | df | Mean Square | F | Sig. | Partial Zeta Squared |
| --- | --- | --- | --- | --- | --- | --- |
| Corrected Model | 17.819^a^ | 9 | 1.980 | 1.104 | .370 | .117 |
| Intercept | .002 | 1 | .002 | .001 | .974 | .000 |
| Gender | .054 | 1 | .054 | .030 | .863 | .000 |
| Age | 1.068 | 1 | 1.068 | .595 | .443 | .008 |
| Smoking Status | .342 | 2 | .171 | .095 | .909 | .003 |
| BMI | .569 | 1 | .569 | .317 | .575 | .004 |
| FEV_1_ (%) | 1.996 | 1 | 1.996 | 1.113 | .295 | .015 |
| VC (%) | 1.023 | 1 | 1.023 | .570 | .453 | .008 |
| FEV_1_/VC | .527 | 1 | .527 | .294 | .589 | .004 |
| F_E_NO (ppb) | .497 | 1 | .497 | .277 | .600 | .004 |
| Error | 134.487 | 75 | 1.793 |  |  |  |
| Total | 542.000 | 85 |  |  |  |  |
| Corrected Total | 152.306 | 84 |  |  |  |  |
| a. R Squared = .117 (Adjusted R Squared = .011) | | | | | | |

TABLE S3

Relationship of asthma exacerbations with patients’ characteristics, and asthma therapy

Anti-asthma treatment with inhaled corticosteroids (ICS), long acting beta-agonist (LABA) and anti-muscarinic (LAMA),and oral leukotrienes-receptor antagonist (LTRA) had no significant influence on exacerbations. The only factor significantly related to the number of exacerbations was the need of oral corticosteroid course.

| Dependent variable: number of exacerbations per year | | | | | | |
| --- | --- | --- | --- | --- | --- | --- |
| Source | Type III Sum of Squares | df | Mean Square | F | Sig. | Partial Eta Squared |
| Corrected Model | 42.595^a^ | 11 | 3.872 | 2.663 | .005 | .230 |
| Intercept | .561 | 1 | .561 | .386 | .536 | .004 |
| LABA | .205 | 1 | .205 | .141 | .708 | .001 |
| ICS-LABA | .389 | 1 | .389 | .268 | .606 | .003 |
| LAMA | .628 | 1 | .628 | .432 | .513 | .004 |
| LTRA | 2.231 | 1 | 2.231 | 1.534 | .218 | .015 |
| ICS score | .682 | 1 | .682 | .469 | .495 | .005 |
| Oral CS | 16.479 | 1 | 16.479 | 11.333 | .001 | .104 |
| Gender | .265 | 1 | .265 | .182 | .670 | .002 |
| Age | 1.989 | 1 | 1.989 | 1.368 | .245 | .014 |
| BMI | 2.358 | 1 | 2.358 | 1.622 | .206 | .016 |
| Smoking Status | 1.358 | 2 | .679 | .467 | .628 | .009 |
| Error | 142.496 | 98 | 1.454 |  |  |  |
| Total | 666.000 | 110 |  |  |  |  |
| Corrected Total | 185.091 | 109 |  |  |  |  |
| a. R Squared = .230 (Adjusted R Squared = .144) | | | | | | |

TABLE S4

Relationship of asthma exacerbations with nutritional status

The only nutritional factor significantly related to the number of exacerbations was vitamin D (25-OHD). Other nutrients had no significant influence on exacerbations The only nutritional factor significantly related to the number of exacerbations was vitamin D (25-OHD). Other nutrients had no significant influence on exacerbations.

| Dependent Variable: number of exacerbations | | | | | | |
| --- | --- | --- | --- | --- | --- | --- |
| Source | Type III Sum of Squares | df | Mean Square | F | Sig. | Partial Eta Squared |
| Corrected Model | 24.286^a^ | 13 | 1.868 | 1.506 | .167 | .372 |
| Intercept | 2.221 | 1 | 2.221 | 1.790 | .190 | .051 |
| 25-OHD | 11.241 | 1 | 11.241 | 9.059 | .005 | .215 |
| Calcium | .412 | 1 | .412 | .332 | .568 | .010 |
| Phosphorus | 1.266 | 1 | 1.266 | 1.020 | .320 | .030 |
| PTH | .069 | 1 | .069 | .056 | .815 | .002 |
| Ferritin | .811 | 1 | .811 | .653 | .425 | .019 |
| Vitamin B12 | .172 | 1 | .172 | .139 | .712 | .004 |
| Magnesium | 1.003 | 1 | 1.003 | .808 | .375 | .024 |
| Folic acid | 1.023 | 1 | 1.023 | .824 | .371 | .024 |
| Gender | .063 | 1 | .063 | .051 | .823 | .002 |
| Age | .000 | 1 | .000 | .000 | .985 | .000 |
| BMI | .087 | 1 | .087 | .070 | .793 | .002 |
| Smoking Status | 1.232 | 2 | .616 | .497 | .613 | .029 |
| Error | 40.948 | 33 | 1.241 |  |  |  |
| Total | 278.000 | 47 |  |  |  |  |
| Corrected Total | 65.234 | 46 |  |  |  |  |
| a. R Squared = .372 (Adjusted R Squared = .125) | | | | | | |

PTH serum parathyroid hormone

TABLE S5

Relationship of vitamin D with exacerbations, lung function tests and ICS dose

Vitamin D was significantly negatively related with the number of exacerbations, but was independent of lung function tests and dose of inhaled corticosteroids (ICS).

| Dependent Variable: Baseline Vitamin D | | | | | | |
| --- | --- | --- | --- | --- | --- | --- |
| Source | Type III Sum of Squares | df | Mean Square | F | Sig. | Partial Eta Squared |
| Corrected Model | 1413.708^a^ | 5 | 282.742 | 5.944 | .000 | .231 |
| Intercept | 492.596 | 1 | 492.596 | 10.356 | .002 | .095 |
| Exacerbations | 863.048 | 1 | 863.048 | 18.144 | .000 | .155 |
| FEV1 (%) | 12.938 | 1 | 12.938 | .272 | .603 | .003 |
| FEV1/FVC | 3.762 | 1 | 3.762 | .079 | .779 | .001 |
| ICS Score | .805 | 1 | .805 | .017 | .897 | .000 |
| Error | 4709.082 | 99 | 47.566 |  |  |  |
| Total | 35489.800 | 105 |  |  |  |  |
| Corrected Total | 6122.790 | 104 |  |  |  |  |
| a. R Squared = .231 (Adjusted R Squared = .192) | | | | | | |

TABLE S6

Relationship of vitamin D with comorbidities

The only comorbidities related with vitamin D were rhinosinusitis and osteoporosis. Other comorbidities, in particular concomitant COPD, had no significant influence on the levels of 25-OHD.

| Dependent Variable: Baseline Vitamin D | | | | | | |
| --- | --- | --- | --- | --- | --- | --- |
| Source | Type III Sum of Squares | df | Mean Square | F | Sig. | Partial Eta Squared |
| Corrected Model | 1083.648^a^ | 14 | 77.403 | 1.382 | .178 | .177 |
| Intercept | 735.467 | 1 | 735.467 | 13.136 | .000 | .127 |
| BMI | 37.588 | 1 | 37.588 | .671 | .415 | .007 |
| Smoking Status | 20.143 | 2 | 10.072 | .180 | .836 | .004 |
| Atopy | 16.022 | 1 | 16.022 | .286 | .594 | .003 |
| Rhinitis | 79.501 | 1 | 79.501 | 1.420 | .237 | .016 |
| Rhinosinusitis | 342.513 | 1 | 342.513 | 6.117 | .015 | .064 |
| Osteoporosis | 383.162 | 1 | 383.162 | 6.843 | .010 | .071 |
| Hypertension | 16.274 | 1 | 16.274 | .291 | .591 | .003 |
| Heart disease | 11.244 | 1 | 11.244 | .201 | .655 | .002 |
| Depression | .880 | 1 | .880 | .016 | .900 | .000 |
| Thyroid disease | 21.333 | 1 | 21.333 | .381 | .539 | .004 |
| OSAS | 18.831 | 1 | 18.831 | .336 | .563 | .004 |
| COPD | 45.036 | 1 | 45.036 | .804 | .372 | .009 |
| GERD | 25.552 | 1 | 25.552 | .456 | .501 | .005 |
| Error | 5039.143 | 90 | 55.990 |  |  |  |
| Total | 35489.800 | 105 |  |  |  |  |
| Corrected Total | 6122.790 | 104 |  |  |  |  |
| a. R Squared = .177 (Adjusted R Squared = .049) | | | | | | |
